# Intelligent Multisensor Prodder for Training Operators in Humanitarian Demining

**DOI:** 10.3390/s16070965

**Published:** 2016-06-24

**Authors:** Roemi Fernández, Héctor Montes, Manuel Armada

**Affiliations:** 1 Centre for Automation and Robotics (CAR) CSIC-UPM, Ctra. Campo Real, km. 0,200, La Poveda, Arganda del Rey, Madrid 28500, Spain; hector.montes@car.upm-csic.es (H.M.); manuel.armada@csic.es (M.A.); 2 Facultad de Ingeniería Eléctrica, Universidad Tecnológica de Panamá, Panamá 0819, Panamá

**Keywords:** intelligent feedback prodder for training, force exerted, prodder’s angle, humanitarian demining

## Abstract

Manual prodding is still one of the most utilized procedures for identifying buried landmines during humanitarian demining activities. However, due to the high number of accidents reported during its practice, it is considered an outmoded and risky procedure and there is a general consensus about the need of introducing upgrades for enhancing the safety of human operators. With the aim of contributing to reduce the number of demining accidents, this paper presents an intelligent multisensory system for training operators in the use of prodders. The proposed tool is able to provide to deminers useful information in two critical issues: (a) the amount of force exerted on the target and if it is greater than the safe limit and, (b) to alert them when the angle of insertion of the prodder is approaching or exceeding a certain dangerous limit. Results of preliminary tests show the feasibility and reliability of the proposed design and highlight the potential benefits of the tool.

## 1. Introduction

Unfortunately, after the end of a war, it is quite frequent that the affected populations have to confront the legacy of landmines planted in its territory. Anti-personnel mines, anti-tank mines, cluster munitions, ERW and IEDs can remain active for decades, and hurting or killing indiscriminately any living being that accidentally activates them. The most recent report from the Landmine and Cluster Munition Monitor organization indicates that in 2014 a total of 3678 mine/ERW casualties were recorded, making an average of 10 casualties per day and a 12% increase from 2013. From this total, the 80% were civilians, and 39% were children. Taking into account that in many states and areas, numerous casualties go unrecorded, it is possible to affirm that the true casualty figure is significantly higher [1].

Apart from the human casualties, the presence of landmines also produces negative economic effects, as it denies access to the affected areas and their resources, causing deprivation and social problems among the affected populations. Therefore, elimination of antipersonnel mines is a vital requirement for the recovery of the affected regions [2]. Humanitarian demining, unlike the military demining, where a clearance rate of 80% to 90% is well-accepted [3], requires the complete removal of all mines, with a demining rate of 100%, so that the cleared minefields may be reverted to normal use [4,5,6]. This turns humanitarian demining into a long and difficult process. 

Today, a mine-clearer’s work still depends on metal detectors and prodders, with all the danger this entails. Prodders are mainly used as complement to metal detectors, so that once a possible target has been detected, the prodder allows to locate it precisely in the terrain, providing information on the depth, size, shape and orientation of the target before attempting to safely excavate it. Finding mines with a prodder involves pushing tool into the ground and relaying on tactile feedback to identify an obstruction that maybe a mine [7]. An expert operator is even able to characterize explosives and housing materials with it. Nevertheless, prodding of landmines is a major cause of demining accidents, especially in those countries where the soil is hard or rocky. 

Conventional prodder has been improved from the original soldier’s bayonets to a lightweight, non-magnetic, and wear-resistant instrument consisting of a stainless rod and a stainless or wooden handle. There are several commercial models that vary in the style of the handle, the length and the hand protection. In addition, there have been several attempts to enhance conventional prodders with advanced functionalities. For instance, DEW Engineering and Development Ltd manufactured for a brief period a prodder with an ability to discriminate between plastic, rock and metal. The SmartProbe^TM^ (DEW Engineering and Development Ltd., Ottawa, Canada) was based on the use of an acoustic pulse to characterize the material under contact. However, several shortcomings in the ergonomic design, ruggedness and performance, discouraged the acquisition of the SmartProbe^TM^ for field use [8]. 

In the scientific and academic community, there are also several relevant prototypes under research. First solutions included the integration of a miniature ultrasonic sensor capable of materials characterization into the hand probe [9,10]. The tip of the prodder was used as a waveguide for transmitting the ultrasonic pulse and receiving reflected energy from the examined object. Recognition among diverse object was performed by using statistical analysis of large number of samples collected from different objects under varying conditions. However, more recent prototypes are based on PZTs and accelerometers, such as the device proposed in [11]. This prodder vibrates a buried target via the PZT actuator. Then, depending on the stiffness of the target, specific accelerations return through the stick and are measured by the accelerometer mounted on the prodder. Thus, the stiffness of the buried target being in touch with the prodder tip can be determined by analyzing the acceleration signals. In [12,13], the authors present a novel smart prodder that is capable of recognizing the material of the suspected object. This prodder consists of: (i) a pair of piezoelectric transducers, used as actuator and sensor; (ii) a force sensor to guarantee a constant application force, and consequently, a good repetitiveness of the piezoelectric response; and (iii) an inclinometer to improve the reliability of the contact. A prodder system based on tactile augmentation is described in [14]. The device is equipped with an accelerometer to sense vibrations and a piezoelectric actuator to amplify the measured acceleration and to generate the tactile sensation to the operator. A different solution is presented in [15,16], where authors state that basic parameters of landmines buried in sandy desert can be accurately estimated according to the contact pressure sensed by a rolling cylinder and by using a PNN. The rolling cylinder is endowed with a with fixable pressure mat, and is pushed to roll over the sand with a constant pressure (less than the activation pressure of any landmine). In consequence, a pressure distribution is generated on the sand surface due to the difference between the Young’s modulus of the sand and the landmine. This pressure distribution is measured by the rolling cylinder and then utilized as input for the PNN, which is responsible of the landmine characterization.

The design of innovative prodders may contribute to increase the rate of mines detected but does not definitively increase the safety of the deminers. Training is then one of the most crucial aspects in order to improve the safety and effectiveness of the landmine detection activities performed by human operators [17]. However, in the literature there is not any study that has been devoted to the improvement of training tools utilized during prodding activities. Prodding has the disadvantage that unlike other aspects of training, the use of excessive force cannot be detected simply by a supervisor observing the trainee. Some studies [18] involving field measurements of the force exerted by the operators showed that deminers, and even senior training staff, had no real idea of the force they were using and consistently underestimated the force they were exerting by large amounts. Therefore, to alleviate this situation, this paper presents an intelligent prodder that provides useful information to the deminers in two critical issues: (a) the amount of force exerted on the target and if it is greater than the safe limit and, (b) to alert them when the angle of insertion of the prodder is approaching or exceeding a certain dangerous limit. In this way, the tool will contribute to improve the deminers’ competencies and consequently, will help to reduce the number of demining accidents during close-in detection tasks. The work presented here has been carried out within the framework of the TIRAMISU project, funded by the European commission in the 7th Framework Programme.

The rest of the paper is organized as follows. Section 2 describes the design and implementation of the proposed intelligent multisensor prodder for training. Section 3 presents the results obtained from the experimental tests carried out with a prototype of the tool. Section 4 discusses the main results of this work and finally, Section 5 summarizes major conclusions. 

## 2. Materials and Methods

The first step accomplished for the design and development of the proposed tool was the analysis of the present status of training programs held in humanitarian demining for close-in detection tasks, the current role of training tools, the identification of the current problems confronted during the training sessions, and the gathering of the requirements and needs expressed by the end-users. The compiled information was based on both findings from literature and on dedicated interviews and workshops with representatives of fifteen different institutional and private organizations concerned with training for humanitarian demining. International standards (IMAS 06.10), guidelines (CWA 15465), and national standards (NMAS) outline training contents and curricula. Close-in detection and disposal are well covered in the existing standards and regulations for EOD training, though particularly the duration of trainings varies considerably. The equipment and material used during close-in detection training resembles the ones utilized in actual operation as much as possible. Therefore, metal detectors and prodders are the tools most commonly used. However, an analysis of accidents in humanitarian demining shows that 37% of them could have been avoided by better training in close-in detection methods. Thus, after analyzing all the compiled information, the key teaching points that were identified for prodders are the following [19]:

- The prodder shall be inserted into the soil using an angle less than 30°–45° (this limit angle varies depending on local conditions of soil) and every 2–3 cm for each trial, so it can hit the mine laterally. A greater angle could be unsafely, since the detonator of the mine could be achieved by the prodder.
- Probing shall be done softly and gradually so a fuse would not be activated due to an excessive pressure.
- In order to conduct a complete and safe search and marking of the spots where mines and UXO have been found, probing frequency is as follows:
  ○ 4–5 probes on 1 dm^2^ when looking for antipersonnel mines
  ○ 15–20 probes on 1 m^2^ when looking for antitank mines.
- Prodding needs a lot of experience and requires particular skill in hard, stony ground.

Thus, an intelligent multisensory system that provides information about the amount of force exerted, and alerts deminers when the prodder’s angle is approaching or exceeding a certain limit is proposed for the improvement of training tasks and consequently for reducing demining accidents [20]. The proposed tool consists of an instrumented prodder, a USB DAQ module, an electronic module for signals conditioning and a HMI. All basic parts of the instrumented prodder (sensors, the rod with the sharp spike, the handle and the extension) are separable with the ability of replacing different extensions in order to obtain different versions of the prodder, depending on the demining training needs.

**Figure 1:**
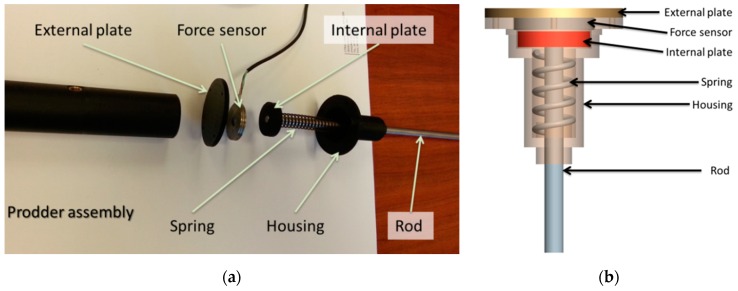
Custom-made installation of the load cell: (**a**) Real disassembled view of the different elements that compose the described design; (**b**) Detailed drawings of the custom-made installation.

For the design of the instrumented prodder, two main types of sensors have been selected and evaluated: a compression load cell and a wireless IMU. Table 1 summarizes the main technical specifications of the selected compression load cell, whereas Figure 1 shows the custom-made installation that has been designed to embed this compression load cell in the prodder. It is possible to see that the force sensing capability has been incorporated in the connection between the handle and the rod.

As in the first 10% of the full scale of operation, the sensor has a non-linear behaviour, a mechanism that preloads the sensor has been incorporated. This simple mechanism consists of a spring and two plates that enclose the sensor. In this way, the sensor will be functioning always in its linear region of operation. The shape and the dimensions of the selected load cell allows and easy adaptation and installation to the currently used prodders, and provides proper reliability, sensitivity and resolution for the required application. 

Table 2 gathers the main technical characteristics of the IMU that was also installed in the prototype. This unit is able to measure the following parameters:

- Pitch, roll and yaw angles.
- Angular and linear velocities in the 3 axes of the Cartesian coordinate system.
- Accelerations in the 3 axes of the Cartesian coordinate system.

**Figure 2:**
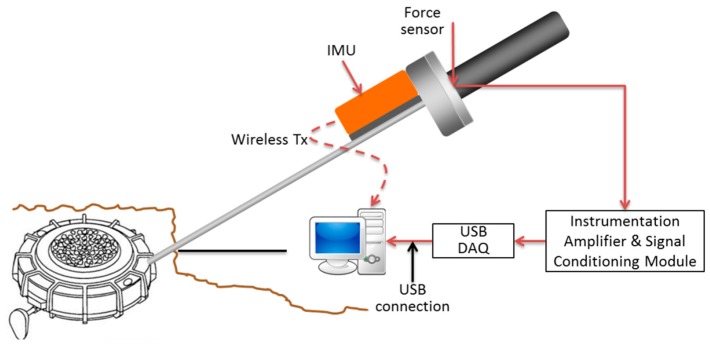
Main components of the proposed tool.

**Figure 3:**
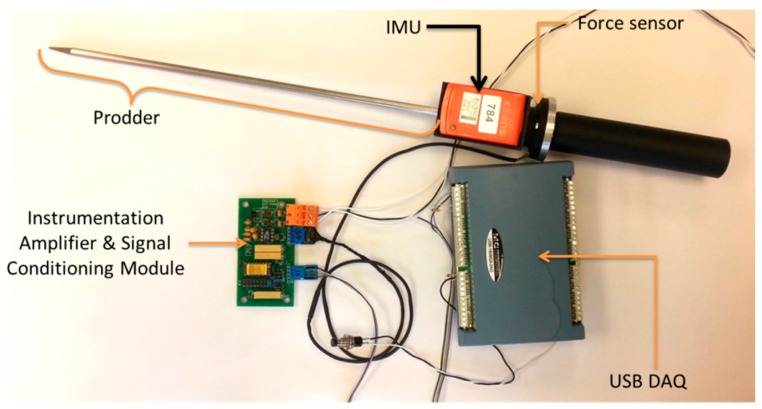
Main components of the proposed tool.

The electronic module for signals conditioning is responsible of filtering and amplifying the force sensor output in order to meet the requirements of the next stage, in which the DAQ module converts the resulting analogic signal into a digital one for further processing. The USB DAQ module is connected to a PC where the HMI is installed, so that the HMI can be able to process, record and display the trainee data. The sampling frequency was chosen to be 100 Hz for the IMU signals and 500 Hz for the force signal. Figure 2 shows a block diagram of the intelligent feedback prodder with the IMU and the force sensor, the electronic module for signals conditioning and the DAQ module. Figure 3 displays a real photo with the main components of the proposed tool. 

In addition, it is important to mention that the instrumented prodder is in conformity with the technical requirements stipulated in books of rules and regulations for devices and equipment used in Humanitarian Demining [21]. These technical requirements are the following:

- The stick with the sharp spike is made of stainless steel—prochrome (antimagnetic), the extensions are made of aluminum alloy, and the handle made of steel coated with rubber or aluminum alloy.
- The length of the stick with sharp spike is at least 400 mm.
- The thickness of the stick with sharp spike (diameter) is at least 8 mm.
- The length of the handle is at least 90 mm.
- The length of the extension is at least 400 mm.
- The outer diameter of the extension is at least 20 mm.
- The total mass of the feedback prodder is less than 600 g.
- The prodder has a rugged, sealed construction for field-portability.
- The feedback prodder for training is cost-effective, reliable and safe.

On the other hand, the HMI is responsible of collecting the data acquired by the sensors of the instrumented prodder, processing, analyzing and monitoring the measured performance variables, and presenting the essential information required during the training sessions, including the activation of relevant alarms when at least one of the following conditions is fulfilled:

- The operator exceeds a pre-defined maximum force. The value of this maximum force will depend on the soil type, the soil conditions and the kind of target.
- The angle of insertion of the prodder exceeds 45°, since that could activate the mine detonation. Nevertheless, this limit angle can be adjusted depending on the local conditions.

The HMI design process consisted of three differentiated phases:

- Assessment of HMI needs and requirements. Thus, the design process started with a review of the SOP, the functional needs, system requirements and the objectives of the training activities.
- Design of the graphical user interface. Thus, main functionality and key components of the HMI were identified and defined, and a draft version of the HMI was developed.
- Finally, system capabilities were evaluated to ensure that the HMI design would fulfil all the needs and requirements identified for it.

**Figure 4:**
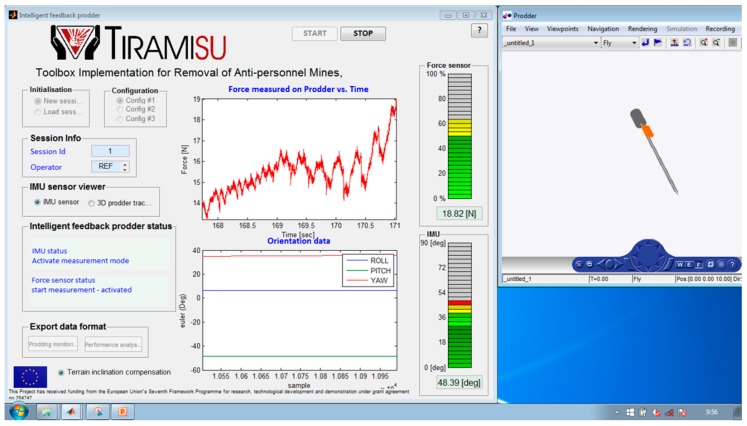
HMI for the intelligent feedback prodder.

Figure 4 shows a screenshot of the designed HMI. The HMI console is the principal mechanism though which instructor interacts with and controls the performance of trainees. Some important sections of the HMI console are described below [22].

### 2.1. Configuration 

This section provides the possibility of loading different configuration files for modifying the objectives of the training session (reference values for the force exerted and the insertion angle of the prodder) according to the soil type and the kind of target to be detected (see Figure 4). Thus, for instance, in highly compact, hard or rocky soil, objectives are set to achieve an approach at a shallow angle. Force reference values are defined taking into consideration the type of mine, its dimensions and its activation load or activation pressure [16,23,24]. 

### 2.2. IMU Sensor Viewer

This section contains two radio buttons that are not mutually exclusive (see Figure 4). The first one, named “IMU sensor” opens and displays a window for calibrating the IMU. The second one, named “3D prodder tracking” opens and displays the Prodder Monitoring window, which is described below.

### 2.3. Prodder Monitoring

**Figure 5:**
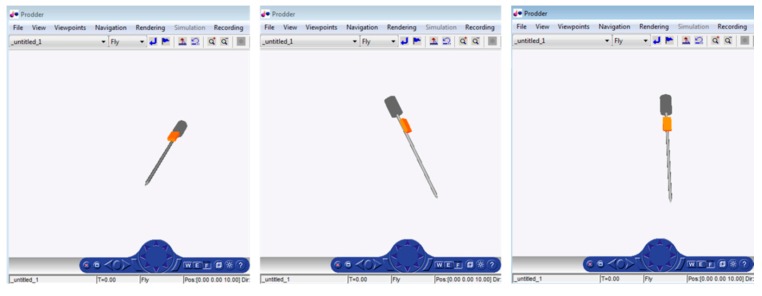
Different snapshots from the prodder monitoring.

This VRML graphic reconstructs in real time the orientation of the prodder carried out by the human operator. The graphic enables the instructor to check if each prodding is being performed with a proper angle of insertion. Figure 5 shows different snapshots from the prodder monitoring. 

### 2.4. Force and Orientation Graphics

**Figure 6:**
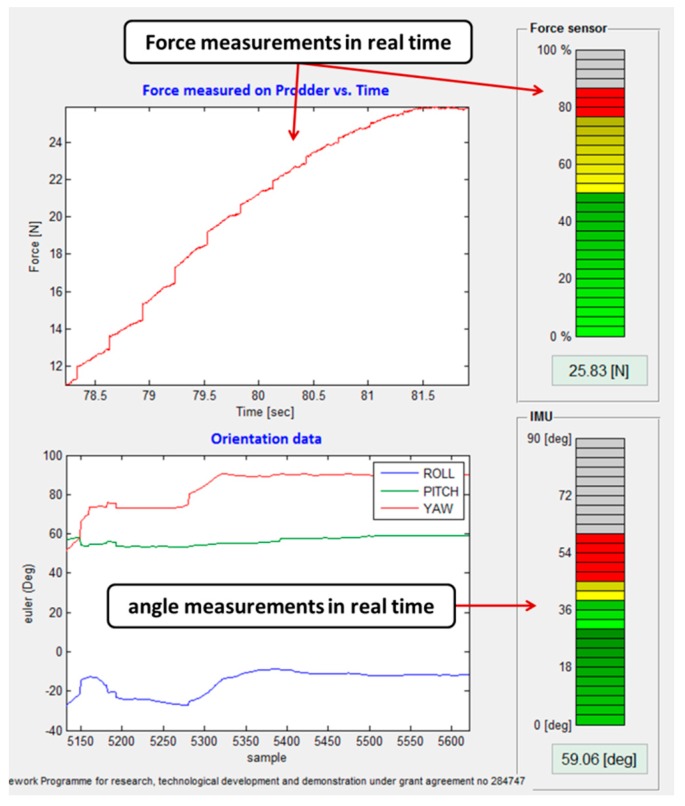
Close-up view of the force and angle data provided by the HMI.

These two graphics display in real time the force exerted by the human operator while prodding, in N, and the roll, pitch, yaw angles in degrees, describing the orientation of the prodding during the training session (see Figure 6).

### 2.5. Force and Angle Data

In this section, data acquired by the intelligent feedback prodder is turned into useful information that will help the instructor to monitor the current situation. Two performance variables are utilized for this purpose: the force exerted by the human operator while prodding in N, and the angle of insertion of the prodder in degrees, which is given by the pitch angle measured independently of the roll and yaw angles. It is also important to mention that this angle is measured with respect to the ground by incorporating a second IMU that is placed on the terrain where prodding is being conducted, and by indicating this option in the designed HMI (see Figure 4). In this way, the second IMU measures the inclination of the terrain, given once again by the pitch angle, and this measurement is then utilized in the HMI for compensating the resulting angle of insertion of the prodder that is provided to the user. Analogic representation of force and angle values, indicating their position relative to normal, abnormal and alarm conditions are displayed on the graphical user interface. The alarms included for each variable will enable the operator to quickly detect values outside the safety range, so he wouldn’t have to relay in his memory and mentally compare each value to its corresponding defined range to discover deviations of trainee objectives. In addition, colors are utilized in two bar graphics to indicate if the performance is holding or not within the training objectives: green is used for indicating that all the evaluated variables are within the training objectives, yellow for warning that the values are starting to deviate from the goals and red for values out of the defined safety ranges (see Figure 6). 

Therefore, with the proposed easy-to-use interface, the instructor is capable of:

- Monitoring the performance variables of the operator, which are the force exerted and the angle of insertion of the prodder.
- Recording all the acquired information in a database.
- Assessing the performance operation of the trainees.
- Recording long data-runs without data loss.

## 3. Results

**Figure 7:**
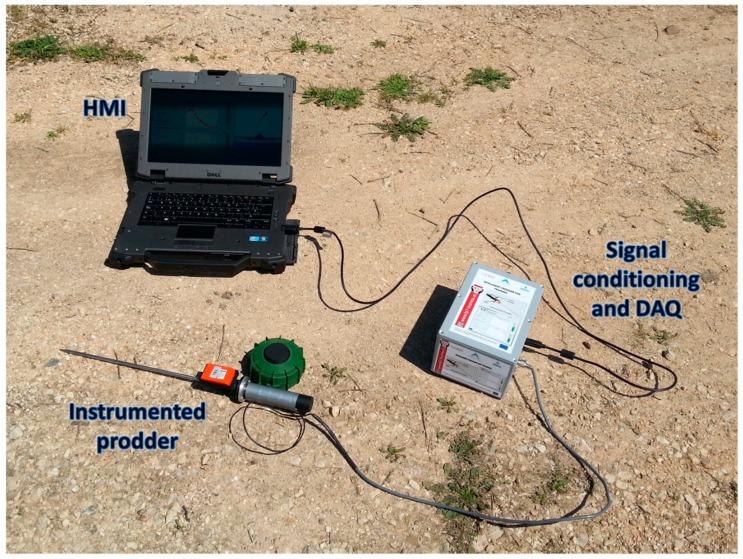
Prototype of the propose tool used during the experimental stage.

Several experimental tests have been carried out under controlled conditions in order to evaluate the technical performance of the proposed tool. Figure 7 shows the prototype that has been used during the tests. This prototype consists of an instrumented prodder, a box that contains the signal conditioning module and the DAQ module, and a HMI. The HMI is installed on a fully rugged laptop that has autonomy of approximately 3 h working continuously. The box that contains the electronic modules has autonomy of 6 h. In addition, the instrumented prodder has been designed in such a way that its length, thickness and total mass are in conformity with technical requirements stipulated in books of rules and regulations for equipment used in Humanitarian Demining. Therefore, the proposed training tool can be easily deployed on the field (see Video S1).

**Figure 8:**
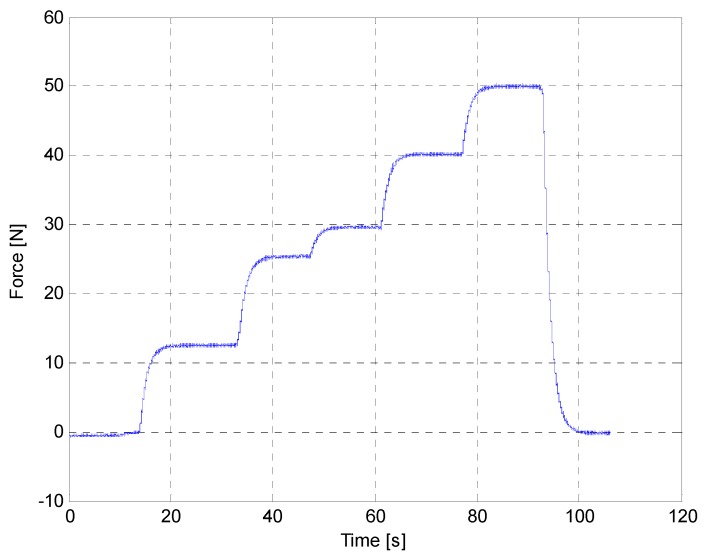
Measurements obtained with the force sensor of the instrumented prodder.

**Figure 9:**
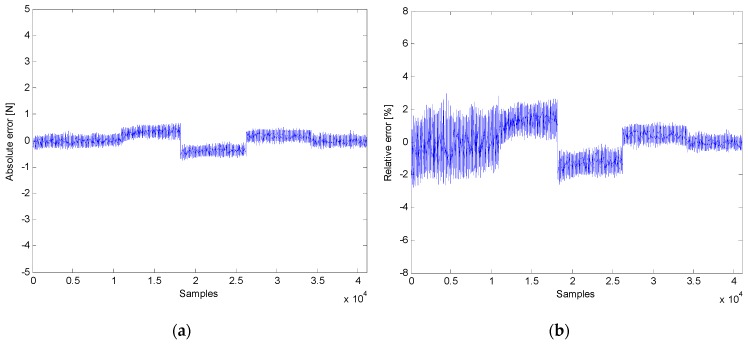
Force measurement errors: (**a**) Absolute error; (**b**) Relative error.

The first part of the experimentation was devoted to the assessment of the force feedback provided by the intelligent prodder. Figure 8 displays several force measurements obtained with the instrumented prodder. In this test, the prodder was located vertically and was gradually loaded with masses of 1.25 kg, 2.5 kg, 3 kg, 4 kg and 5 kg. The mean values measured by the sensory system were 12.5 N, 25 N, 30 N, 40 N and 50 N, approximately. Figure 9 shows the absolute and the relative errors for the obtained force measurements. Thus, results provided by the intelligent feedback prodder demonstrate that the force sensor has an accurate dynamic response. 

**Figure 10:**
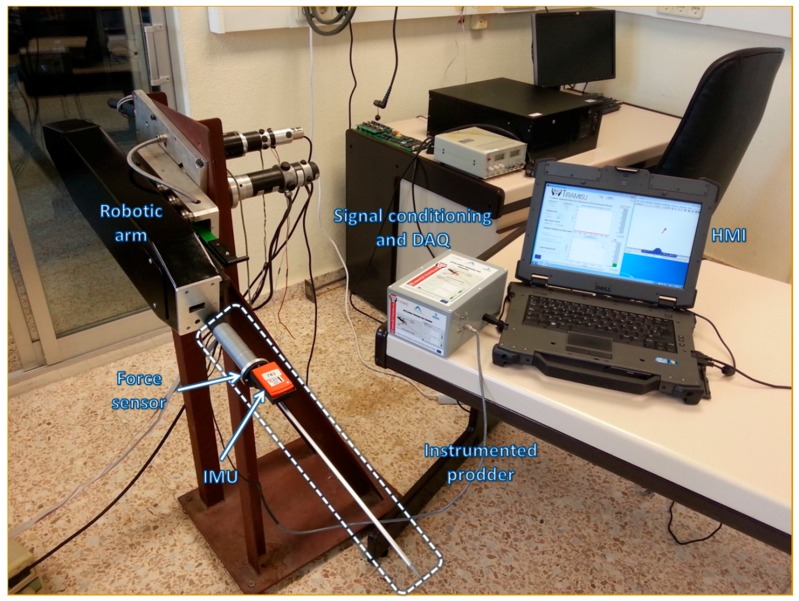
Intelligent feedback prodder installed in the robotic arm for the second part of the experimentation.

For the second part of the experimentation, a robotic arm was utilized to handle the intelligent feedback prodder and to carry out repetitive and exhaustive robotic testing (see Video S2). In this way, it was possible to control the angle in each prodding with high accuracy and to use this information as ground truth data during the comparison with the results measured by the intelligent sensory system. Figure 10 shows the set-up utilized for this experimental phase, which consists of the instrumented prodder installed on the robotic arm and connected to the HMI through the signal conditioning and DAQ module and the wireless LAN network connection implemented between the IMU and the HMI.

**Figure 11:**
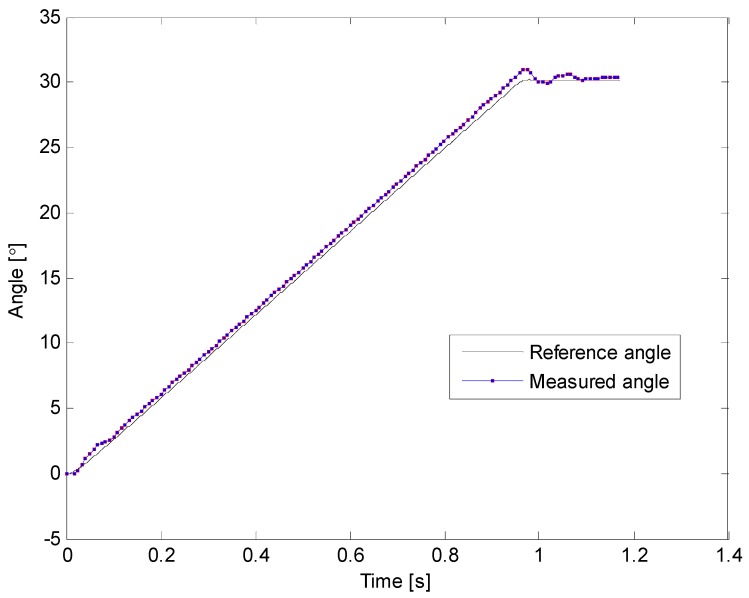
Inclination angle vs. time—first trial.

**Figure 12:**
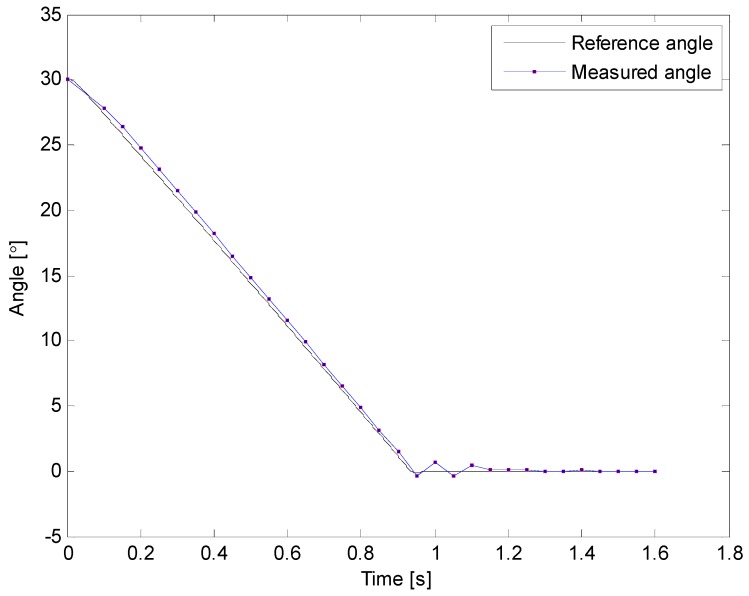
Inclination angle vs. time—second trial.

Figure 11 and Figure 12 illustrate results of two of the experimental tests that were carried out. Black lines represent the reference angle trajectory programmed for the first degree of freedom of the robotic arm, which corresponds to the movement of the shoulder, whereas the red squares plotted on the red lines represent the measurements acquired with the sensory system of the proposed prodder. On these examples, the robot was programmed to move its shoulder 30° from the horizontal position, and once this position is achieved, come back to the original configuration. Note also that on Figure 11, sampling rate was set to 120 Hz for the measurement of the prodder’s angle, while in Figure 12 the sample rate was decreased to 20 Hz. In both cases, the angle tracking provided by the intelligent feedback prodder exhibits a high accuracy.

**Figure 13:**
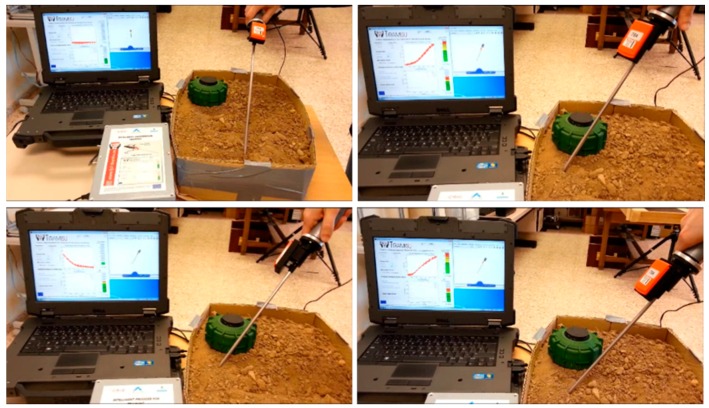
Scenario for the third sequence of experiments.

**Figure 14:**
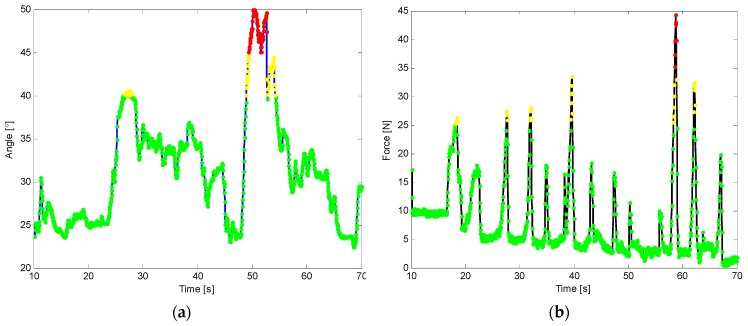
Activation of alarms: (**a**) Inclination angle measurements; (**b**) Force measurements.

The last set of trials was devoted to verifying the correct activation of alarms when the operator exceeds the pre-defined maximum force, or when the angle of insertion of the prodder exceeds 45°, which can activate the mine detonation. The result presented corresponds to a test where the prodder is inserted in the terrain until a buried metallic piece is found. Figure 13 shows the scenario of the experiment, whereas Figure 14 presents the obtained results. The blue solid line in Figure 14a represents the acquired inclination angle of the prodder, black solid line in Figure 14b represents the exerted force, and finally, dotted lines in Figure 14a,b represent the behavior of the alarms for the intelligent feedback prodder, in such a way that green color indicates that values are within the training objectives, yellow color warns that values are starting to deviate from the goals and red colors informs that values are out of the defined safety ranges. This feedback is crucial to teach trainees to establish good working habits. For instance, if there is a chance that maximum force is exceeded during training session in highly compact, hard, or rocky soil, trainee should learn to apply some techniques that are commonly used in practice to avoid accidents, such as: (i) softening the ground before prodding, (ii) excavating, or (iii) penetrating soil with low force by slowly pressing the tip of the prodder into the ground and loosen pebbles with a slow twist of the wrist.

## 4. Discussion

**Figure 15:**
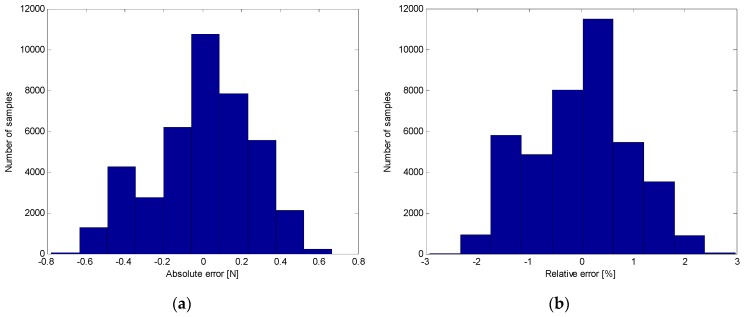
Distribution of force measurement errors: (**a**) Absolute error; (**b**) Relative error.

Gathering together the quantitative results obtained from the experimental tests presented in the previous section, it is possible to highlight the high accuracy of the proposed intelligent sensory system to estimate in real-time the force exerted by the operator as well as the angle of inclination of the prodder. Figure 15 shows the distribution of the absolute and relative errors of the force measurements acquired in the experimental stage. Results demonstrated that the proposed tool is able to provide information about the tip forces in the rod direction with a maximum absolute error of ±0.7 N and a maximum relative error of ±3% in dynamic conditions. Thus, the force feedback provided by the training tool will be very useful to teach trainees what a safe prodding force feels like, in order to establish good working habits.

**Figure 16:**
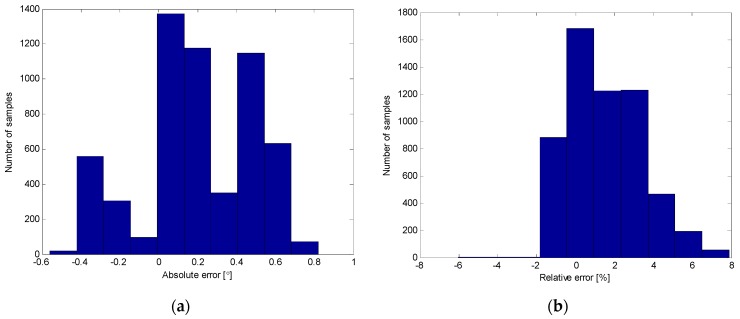
Distribution of measurement errors from prodder’s inclination angle: (**a**) Absolute error; (**b**) Relative error.

Figure 16 shows the distribution of the absolute and relative errors of the prodder’s angle measurements acquired during the experimental tests. Results demonstrated that the proposed tool is able to provide information about the angle of insertion of the prodder with a maximum absolute error that ranges from −0.6° to 0.8° and a maximum relative error that goes from −6% to 7.9% in dynamic conditions. These highly accurate measurements of the prodder’s angle will be very important to aware deminers when they are approaching or exceeding a certain limit that can drive to the mine detonation.

Lastly, the third phase of the experimentation confirmed that alarms provided by the HMI are activated in 100% of the cases. These alarms were designed for alerting the trainee when he/she is approaching or exceeding a certain limit that can drive to the mine detonation, and thus, play a crucial role during training activities.

During the experimental tests, the HMI also demonstrated its versatility and reliability in gathering, analyzing, presenting and consolidating the information acquired with the instrumented prodder that has been especially conceived and implemented for interacting with this application. The friendly graphic user interface presents the data received in an efficient format, maximizing the instructor’s ability for monitoring, processing and evaluating the trainee performance, and consequently, reducing the total cognitive load required. 

Despite all the advantages provided by the proposed tool, and despite its design and implementation have been carried out taking into consideration the needs and the requirements of the end-users, its introduction in the current training programs can be hindered by the strong rootedness to the traditional methods. One of the largest barriers to the adoption of instrumented prodders by the demining community is the rigid adherence to existing operating procedures. The demining community perceives the current operating procedures as safe and refuses the introduction of new equipment that does not have pre-existing safety record. Hence the importance of disseminating the obtained results, with the aim of increasing the degree of acceptance of the end-users and facilitate the adoption of the proposed tool. In this way, instructions will be based on scientific knowledge rather than on personal introspections and intuitions.

## 5. Conclusions

This paper addressed the design, implementation and technical validation of a novel intelligent multisensory system for improving the training activities of humanitarian demining operations carried out with prodders. The main objective of the training tool is to provide force and angle feedback to teach trainees how a safe prodding should be conducted, in order to establish good working habits, and consequently contribute to the reduction of the number of accidents during the practice of this risky and dangerous task. An outline of the main features, functions and components of the system has been described in detail. The proposed tool consists of a prodder instrumented with a custom-made force sensor and an IMU, an electronic module for signals conditioning, a DAQ module, and a HMI. All basic parts of the instrumented prodder are separable and easily interchangeable in order to obtain different versions of the prodder, depending on the demining training needs. Experimental tests demonstrated that with the proposed training tool it is possible to determine in real-time and with high accuracy, the tip forces in the rod direction as well as the angle of inclination of the prodder. Thus, the proposed tool is able to provide useful information about the amount of force exerted and alert deminers when the angle of insertion of the prodder is approaching or exceeding a certain limit while they are prodding. In addition, the designed HMI has the advantage of providing an overview of the entire operation conducted with the prodder and a limited number of well-defined alarms. In this way, the instructor or the trainee will be able to see the entire operation almost at-a-glance. Therefore, the proposed tool will improve the instructor’s ability for monitoring, processing and assessing the performance data of the training, reducing the total cognitive load. The intelligent multisensor prodder proposed in this work represents a significant advance in the current state-of-the-art, since it enables the development and implementation of training sessions based on scientific analysis of the problem and the formative and summative assessment of trainees, rather than on personal introspection and intuitions of the training designers.

Future work will be directed to design and implement a RF module that allows us to eliminate the cable that connects the force sensor installed on the instrumented prodder with the box that contains the signal conditioning module, providing enhanced flexibility to the proposed tool. In addition, validation tests will be conducted in real scenarios with demining end-users in order to confirm the benefits of the intelligent feedback prodder for training. 

## Figures and Tables

**Table 1:** Main technical specifications of the compression load cell.

**Non-linearity**	**Hysteresis**	**Thermal Zero Shift**	**Thermal Sensitivity Shift**
±1% FSO	±1% FSO	±2.5 mV/50 °C	±2.5%/50 °C
**Deflection at “FS”**	**Operating Temperature**	**Thickness**	**Diameter**
<0.013 mm nom.	(−40 to 120) °C	3.81 mm	25.4 mm

**Table 2:** Main technical specifications of the IMU.

Parameters	Orientation Performance	Parameters	Angular Velocity	Acceleration
Dimensions	pitch, roll, yaw	Dimensions	3 axes	3 axes
Full scale	±180°	Full scale	±120°/s	±1600 m/s^2^
Angular resolution	0.05°	Linearity	0.1% FS	0.2% FS
Dynamic accuracy	2° RMS	Alignment error	0.1°	0.1°

## Supplementary Materials

The following are available online at http://www.mdpi.com/1424-8220/16/7/965/s1, Video S1: Intelligent feedback prodder for training, Video S2: Robotic testing.

## Abbreviations

The following abbreviations are used in this manuscript:

ERW	Explosive remnants of war
IED	Improvised explosive device
PZT	Piezo-electric transducer
PNN	Perceptron Neural Network
TIRAMISU	Toolbox implementation for removal of antipersonnel mines, submunitions and UXO
UXO	unexploded ordnance
IMAS	International Mine Action Standards
CWA	CEN workshop agreement
CEN	European Centre for Standardization
NMAS	National mine action standards
EOD	explosive ordnance disposal
FSO	Full scale output
FS	Full scale
USB	Universal serial bus
HMI	Human machine interface
IMU	Inertial measurement unit
VRML	Virtual reality modelling language
DAQ	Data acquisition
LAN	Local area network

## References

[1] International Campaign to Ban Landmines—Cluster Munition Coalition Landmine Monitor 2015. http://www.the-monitor.org/media/2152583/Landmine-Monitor-2015_finalpdf.pdf.

[2] Habib M.K. Mine Clearance Techniques and Technologies for Effective Humanitarian Demining. http://www.jmu.edu/cisr/journal/6.1/features/habib/habib.htm.

[3] Rosengard U., Dolan T., Miklush D., Samiei M. Humanitarian Demining Nuclear Techniques May Help the Search for Landmines. https://www.iaea.org/sites/default/files/publications/magazines/bulletin/bull43-2/43205031619.pdf.

[4] Habib M.K. Humanitarian demining mine detection and sensors. Proceedings of the 2011 IEEE International Symposium on Industrial Electronics.

[5] Hussein E.M., Waller E.J. (2000). Landmine detection: The problem and the challenge. Appl. Radiat. Isotopes.

[6] Shimoi N. (2002). Technology for Detecting and Clearing Landmines.

[7] Smith A. Understanding the Use of Prodders in Mine Detection. http://www.jmu.edu/cisr/journal/18.1/notes/smith.shtml.

[8] Alternatives for Landmine Detection. https://www.rand.org/content/dam/rand/pubs/monograph_reports/MR1608/MR1608.pref.pdf.

[9] Antonic D. Analysis and Interpretation of Ultrasonic Prodder Signal. Proceedings of the MATEST 2003―Achievements & Challenges.

[10] Stepanic J., Maric G., Schauperl Z. Improving Integration of Ultrasonic Sensor and Hand Probe. Proceedings of the 9th European Conference on Non Destructive Testing.

[11] Ishikawa J., Iino A. Experimental test and evaluation of an active sensing prodder. Proceedings of the 8th International Symposium “Humanitarian Demining 2011”.

[12] Baglio S., Cantelli L., Giusa F., Muscato G., Noto A. The development of an inteligent manual prodder for material recognition. Proceedings of the 11th International Symposium “Mine Action 2014”.

[13] Baglio S., Cantelli L., Giusa F., Muscato G. (2015). Intelligent prodder: Implementation of measurement methodologies for material recognition and classification with humanitarian demining applications. IEEE Trans. Instrum. Meas..

[14] Iwatani A., Shoji R., Ishikawa J. Development of prodder for humanitarian demining based on tactile augmentation. Proceedings of the 12th International Symposium “Mine Action 2015”.

[15] Ali H.F.M., Fath El Bab A.M.R., Zyada Z., Megahed S.M. Inclination Angle Effect on Landmine Characteristics Estimation in Sandy Desert Using Neural Networks. Proceedings of the 10th Asian Control Conference (ASCC).

[16] Ali H.F.M., Fath El-Bab A.M.R., Zyada Z., Megahed S.M. (2016). Estimation of landmine characteristics in sandy desert using neural networks. Neural Comput. Appl..

[17] Fernández R., Montes H., Salinas C., Santos P.G.D., Armada M. (2012). Design of a training tool for improving the use of hand-held detectors in humanitarian demining. Ind. Robot Int. J..

[18] Gasser R. (2000). Technology for Humanitarian Landmine Clearance. Ph.D. Thesis.

[19] CROMAC Book of Rules and Regulations on the Training and the Competence Examination of the Employees in Humanitarian Demining. https://www.hcr.hr/en/pravilnici.asp.

[20] Fernández R., Montes H., Gusano J., Sarria J., Armada M. Force and angle feedback prodder. Proceedings of the 17th International Conference on Climbing and Walking Robots, CLAWAR 2014.

[21] Jungwirth O. Book of Rules and Regulations on Technical Requirements and Conformity Assessment of Devices and Equipment Used in Humanitarian Demining. https://www.hcr.hr/en/pravilnici.asp.

[22] Fernández R., Salinas C., Montes H., Sarria J., Armada M. Design of a human machine interface for training activities with prodders. Proceedins of the 12th International Symposium Mine Action.

[23] Jaeger Platoon Website. http://www.jaegerplatoon.net/landmines2.htm.

[24] Absolute Astronomy Website. http://www.absoluteastronomy.com/topics/List_of_landmines.

